# High patient satisfaction and good long-term functional outcome after endoscopic calcaneoplasty in patients with retrocalcaneal bursitis

**DOI:** 10.1007/s00167-020-06167-2

**Published:** 2020-07-25

**Authors:** Kim T. M. Opdam, Ruben Zwiers, Joy Vroemen, Inger N. Sierevelt, Johannes I. Wiegerinck, C. Niek van Dijk

**Affiliations:** 1 Department of Orthopedic Surgery, Amsterdam UMC, Amsterdam, The Netherlands; 2 Academic Center for Evidence Based Sports Medicine (ACES), Amsterdam, The Netherlands; 3 Amsterdam Collaboration for Health and Safety in Sports (ACHSS), Amsterdam, The Netherlands; 4 Department of Radiology and Nuclear Medicine, Meander Medisch Centrum, Amersfoort, The Netherlands; 5 Xpert Orthopedie Amsterdam/SCORE (Specialized Center of Orthopedic Research and Education), Amsterdam, The Netherlands; 6 Bergman Clinics, Rijswijk, The Netherlands; 7 FIFA Medical Centre of Excellence Ripoll-dePrado-vanDijk SportClinic, Madrid, Spain; 8 FIFA Medical Centre of Excellence Clinica do Dragao Porto, Porto, Portugal; 9 Department of Orthopedic Surgery, University of Amsterdam, Amsterdam UMC, PO Box 22660, 1100 DD Amsterdam, The Netherlands

**Keywords:** Ankle, Retrocalcaneal bursitis, Endoscopic calcaneoplasty, Outcome

## Abstract

**Purpose:**

The primary objective of this study was to determine the degree of patient satisfaction at a minimum of 5 years of follow-up after endoscopic calcaneoplasty. The secondary objectives were to assess functional outcome measures, pain scores, analysis of bone removal, reformation of exostosis at follow-up and correlation of the size of the exostosis and recurrent or persisting complaints.

**Methods:**

This study evaluated patients who underwent endoscopic calcaneoplasty, between January 1st 2000 and December 31st 2010, for the diagnosis of retrocalcaneal bursitis. The evaluation consisted of PROMs (patient-reported outcome measures), a questionnaire and a visit to the outpatient clinic for physical examination and a standard lateral weight-bearing radiograph of the ankle. Patient satisfaction, functional outcomes and pain scores were measured by use of a numeric rating scale (NRS). Size of the posterosuperior calcaneal exostosis was measured on a standard lateral weight-bearing radiograph using parallel pitch lines (PPL) and the Fowler–Philip angle (PFA).

**Results:**

The response rate was 28 out of 55 (51%) and the median time to follow-up was 101(IQR 88.5–131.8) months. The median satisfaction score for treatment results was 8.5 out of 10 (IQR 6–10). FAOS symptoms 84.5 (IQR 58.0–96.4), FAOS pain 90.3 (IQR 45.1–100.0), FAOS ADL 94.9 (IQR 58.1–100.0), FAOS sport 90.0 (IQR 36.3–100.0) and FAOS QOL 71.9 (IQR 37.5–93.8) and median AOFAS was 100 (IQR 89–100). The median PLL difference between before operation and 2 weeks after the operation was − 4 mm (IQR-6 and -1) and the median PLL difference between 2 weeks after the operation and at follow-up was 1 mm (0–2). The median PFA was 65 (63–69) at baseline, 66.5 (60.8–70.3) 2 weeks after the operation and 64 (60.8–65.3) at follow-up.

**Conclusion:**

Despite the limited response rate, this study shows high patient satisfaction and good long-term functional outcome in patients affected by retrocalcaneal bursitis who underwent endoscopic calcaneoplasty.

**Level of evidence:**

Level IV.

## Introduction

Retrocalcaneal bursitis is characterized by inflammation of the bursa between the superoposterior aspect of the calcaneus and ventral side of the Achilles tendon resulting in painful swelling, medial and lateral to the Achilles tendon at the level of the posterosuperior calcaneal prominence [1, 12]. Inflammation of this bursa was first described by Painter in 1898 and the first surgical treatment was performed and described by Haglund in 1928 [6]. The contributing biomechanical risk factors are a posterosuperior calcaneal prominence, hindfoot equinus, compensated hindfoot varus, compensated forefoot valgus, rigidly plantarflexed first ray, cavus foot and trauma to the apophysis in childhood [21].

Conservative treatment includes orthotics, avoidance of tight shoes, activity modification, use of padding, physical therapy, non-steroidal anti-inflammatory drugs and corticosteroid injections [21, 24]. If conservative treatment fails, surgical treatment can offer a solution, favoring endoscopic surgery over open surgery [8, 18, 28]. Endoscopic procedures have surpassed open techniques in many ways: lower morbidity, less postoperative pain, excellent perioperative visualization of the retrocalcaneal bursal space and Achilles tendon including its insertion, early allowance of functional rehabilitation, shorter recovery time and quick sports resumption in comparison with open techniques [3, 8, 28]. Short-term outcome on endoscopic calcaneoplasty shows good results on patient satisfaction, pain scale and functional outcome scores. However, little is known about the long-term results of endoscopic for patients with symptomatic retrocalcaneal bursitis. In this study, we assessed the results of endoscopic calcaneoplasty at a minimum of 5-year follow-up. Radiographic measurements were performed to analyze the extent of bone removal and to estimate the reformation of exostosis at follow-up. Also, the size of the exostosis and recurrent or persisting complaints were correlated.

It was hypothesized that there is a high patient satisfaction and good functional outcome after endoscopic calcaneoplasty of retrocalcaneal bursitis on the long term.

## Materials and methods

This study was approved by the local medical ethics committee of the Academic Medical Center (reference number 2015_318#) and performed in accordance with the principles of the Declaration of Helsinki and the medical Research Involving Human Subjects Act (WMO).

### Inclusion criteria and operative technique

In this case series, all patients who underwent endoscopic calcaneoplasty in the orthopedics department of the Academic Medical Center between January 1st 2000 and December 31st 2010 for the diagnosis of chronic retrocalcaneal bursitis were identified. Chronic retrocalcaneal bursitis was defined as a clinical inflammation of the retrocalcaneal bursa as shown by MRI or ultrasound, which did not resolve with at least 6 months of conservative treatment (RICE (Rest, Ice, Compression, Elevation) therapy, pain-inducing activity cessation in combination with a change in footwear and NSAID’s (non-steroid anti-inflammatory drugs). The inclusion criteria were diagnosis by physical examination and additional imaging for retrocalcaneal bursitis, age over 18 years, capable of filling out a questionnaire and signed informed consent. Exclusion criteria were pregnant or possibly pregnant patients, inability to understand the patient information and the questionnaires (e.g. mental retardation, language barrier). If treated bilaterally, the subject was excluded. Endoscopic calcaneoplasty was performed using two portal endoscopic calcaneoplasty technique as described by van Dijk et al. [25, 26].

### Study procedures and data collection

All eligible subjects were extracted from the electronic patient file system. All subjects were invited to participate in our study by mail. To enhance response rate, a reminder was sent 2 weeks after sending the first mail. Participation consisted of PROMs, a questionnaire and a visit to our outpatient department. At consultation, physical examination and one standard lateral weight-bearing radiograph of the ankle were performed.

### Outcome measures

The main study parameter was the degree of patient satisfaction measured using a numeric rating scale (NRS) for satisfaction. This is a 11-point scale, where patients are requested to quantify their degree of satisfaction on a scale from 0 (very unsatisfied) to 10 (very satisfied).

The secondary study parameters were the outcomes of the Foot and Ankle Outcome Score (FAOS), Ogilvie–Harris score, NRS for pain, EQ-5D-3L, American Orthopaedic Foot and Ankle Society (AOFAS) Ankle–Hindfoot Scale and additional questions were asked. Radiographic measurements were done to analyze the extent of bone removal and to assess the reformation of exostosis at follow-up. Also, correlations were made between the size of the exostosis and recurrent or persisting complaints.

### The questionnaire

The FAOS is the most appropriate foot and ankle PROM for general foot and ankle problems and consists of 42 items [22, 23, 27]. It consists of five subscales: Pain, Other Symptoms, Function in Daily Living (ADL), Function in Sport and Recreation, and foot and ankle-related Quality of Life (QOL). Standardized answer options are given and each question is scored from 0 to 4. A normalized score (100 indicating no symptoms and 0 indicating extreme symptoms) is calculated for each subscale.

The Ogilvie–Harris score consists of five subscales: pain, swelling, stiffness, limping and activity. Each item is rated as excellent, good, fair, or poor. The lowest grade among the items determines the final score. The Ogilvie–Harris score is not been validated, however, we added the score because of its previous use in other studies and the importance of subjective outcome scores in clinical evaluation.

A NRS is a common and practical method for assessing pain severity on a scale from 0 (indicating no pain) to 10 (indicating worst pain imaginable) [7].

The EQ-5D-3L is a standardized instrument, designed to measure health outcome applicable to a wide range of health conditions and treatments [16]. The questionnaire consists of five dimensions (mobility, self-care, usual activities, pain/discomfort, anxiety/depression). The responses record three levels of severity (no problems/some or moderate problems/extreme problems).

The following questions were added to the questionnaire. If treatment was effective, the following options were given: ‘complaints never reoccurred’, ‘minor residual complaints’, ‘only a temporary effect’ and ‘was ineffective’. Also, patients were asked about the current function and were given the following options: normal, almost normal, abnormal, and very abnormal and asked if they would undergo the operation again in the same situation. To further decrease the chance of missing data, complications were inquired for during the consultation and this was compared to the data extracted from the electronic patient file system.

### Outpatient clinic with physical examination

Range of motion was investigated as well as, swelling, heel/toe walking, pain on palpation at the insertion of the Achilles tendon at the outpatient clinic. Also, the AOFAS was scored, which is a combined patient-reported and physician reported/objective and subjective clinical rating system. The score ranges from 1 to 100 points and is divided into pain, function and alignment. A score of 100 indicates the best outcome [15].

### Radiological measurements

**Fig. 1:**
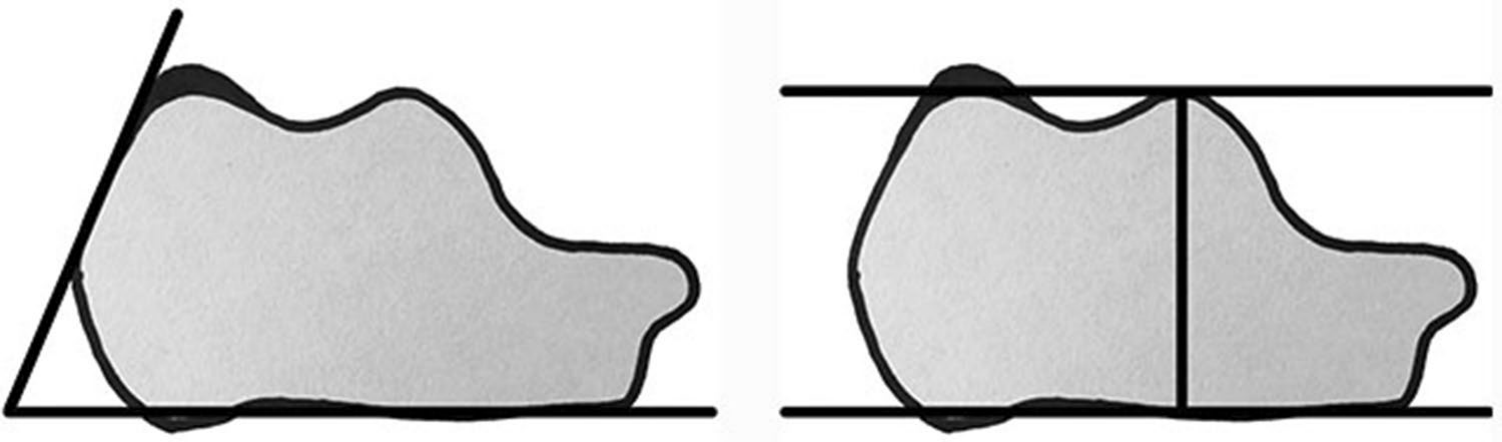
PFA angle (left) and the PPL (right)

Size of the posterosuperior calcaneal exostosis was measured on a standard lateral weight-bearing radiograph using parallel pitch lines (PPL) (See Fig. 1) [20]. Size will be defined as the largest distance between PPL and the calcaneal prominence, measured in mm perpendicular to the PPL.

Also, the Fowler–Philip angle (FPA), which reflects the relation of the inferior calcaneus to the posterior calcaneus, will be measured (see Fig. 1) [5]. This angle is measured on the lateral view of a weight-bearing ankle image, formed between a line tangent to the posterosuperior border of the calcaneus and the calcaneal tuberosity, and a line tangent to the inferior border of the calcaneus (longitudinal axis of the calcaneus). Its normal range is between 44° and 69°. If the FPA is more than 75°, it is diagnostic of enlargement of the posterior aspect of the calcaneus, as seen in retrocalcaneal bursitis [5].

### Statistical analysis

Analyses of outcome data were mainly descriptive. Continuous outcome measures (e.g. FAOS, NRS, EQ-5D) were presented as mean with standard deviation for data with a normal distribution and as median with interquartile range in case of non-normal distribution or ordinal variables (Ogilvie–Harris score). Distribution of continuous variables was assessed using the Kolmogorov–Smirnov test. Outcome data were presented as boxplot. Descriptive data were presented as frequencies with percentages in case of categorical data. Correlations between radiological measurements and outcome data were assessed using Spearman’s rho. Statistical analyses were performed with Statistical Package for Social Sciences (SPSS) version 25.0 (SPSS Inc. Chicago, IL).

## Results

In total, 59 patients, who underwent endoscopic calcaneoplasty in the Academic Medical Center between January 1st 2000 and December 31st 2010 for the diagnosis of retrocalcaneal bursitis were extracted from the electronic patient system. Four patients were treated bilateral and were, therefore, excluded.

**Fig. 2:**
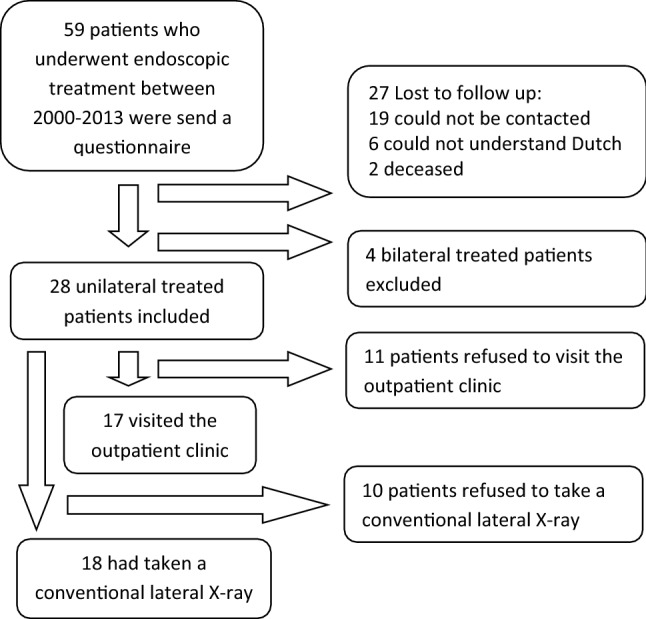
Inclusion flowchart

**Fig. 3:**
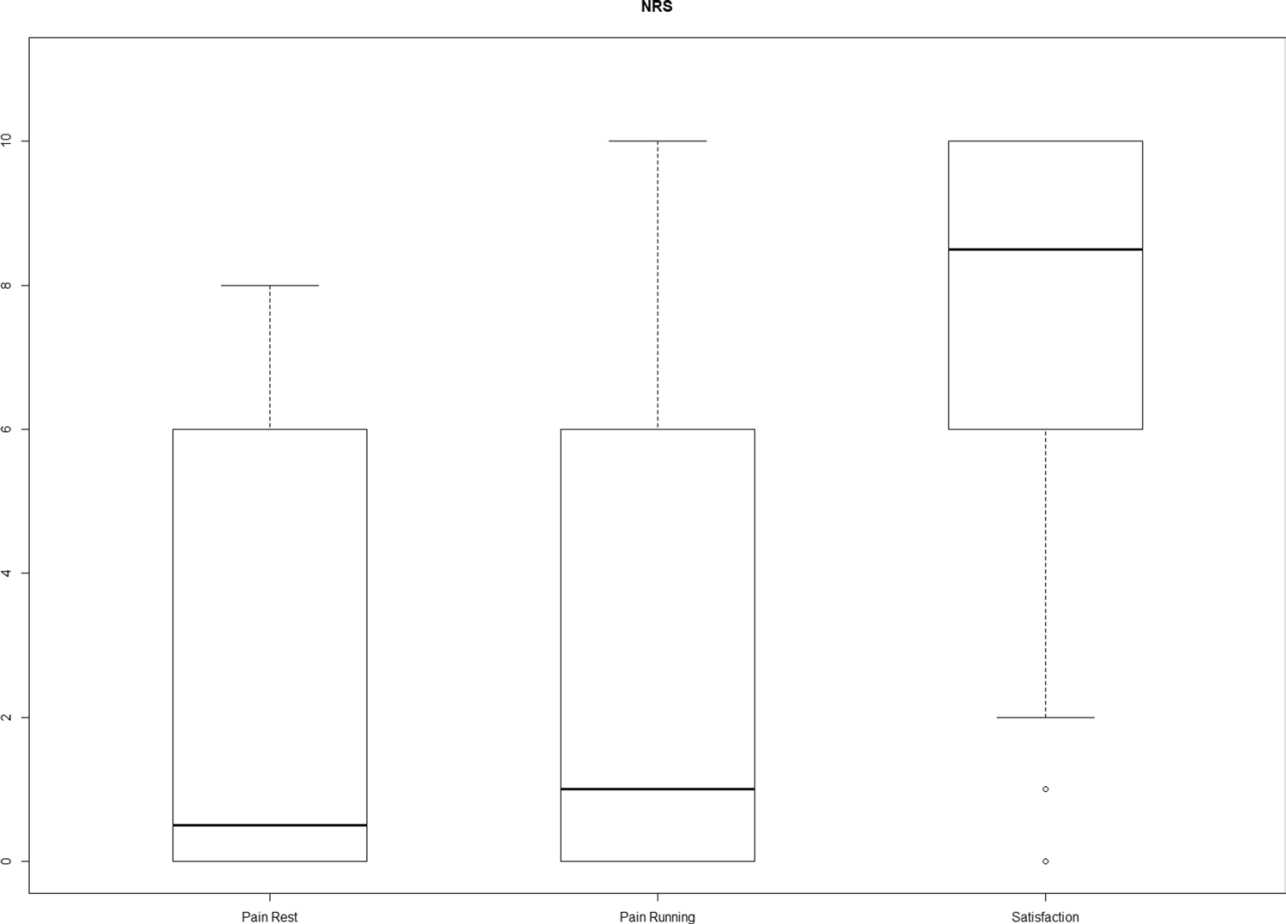
The median NRS satisfaction, NRS pain in rest and NRS pain during running

**Fig. 4:**
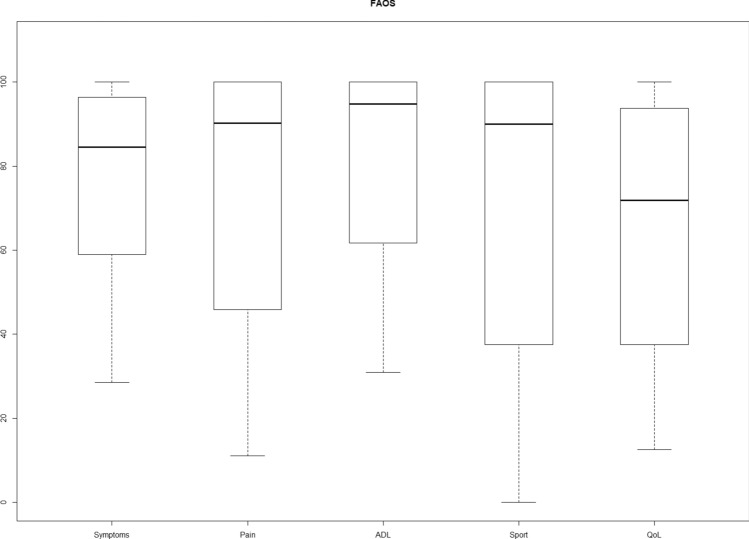
FAOS subscales scores

The response rate was 28 out of 55 (51%). In total, 28 patients were identified as suitable to participate in this study. Of the 28 patients, 17 (67%) patients visited the outpatient clinic and 18 (71%) patients had taken a conventional lateral radiograph of the ankle at outpatient clinic follow-up (See Fig. 2 for the inclusion flowchart). Table 1 presents the characteristics of the participants in the study and non-responders. The median time to follow-up was 101 months (IQR 89–132) and the mean time to follow-up was 113 months (range 69–224, SD 36). Figure 3 presents the median NRS satisfaction of 8.5 (IQR 6–10), NRS pain in rest of 0.5 (IQR 0–6) and NRS pain during running of 1 (IQR 0–7). Figure 4 shows the FAOS subscales scores: symptoms 84.5 (IQR 58.0–96.4), pain 90.3 (IQR 45.1–100.0), ADL 94.9 (IQR 58.1–100.0), Sport 90.0 (IQR 36.3–100.0) and QOL 71.9 (IQR 37.5–93.8). The median EQ-5D-3L was 0.79 (IQR 0.39–1.00). Table 2 shows the outcome of the Ogilvie–Harris score.

**Table 1:** Characteristics of the participants and non-responders

	Responders	Non-responders
	*N* = 28 (100%)	*N* = 27 (100%)
Sex		
Male	13 (46.4%)	19 (70.4%)
Female	15 (53.6%)	8 (29.6%)
Age during operation	Median 47 (IQR 34–52)	Median 37 (IQR28–48)
Body mass index	27.7 (IQR 25.3–30.0)	25 (22.4–28.4)
Side		
Left	11 (39.3%)	14 (51.9%)
Right	17 (60.7%)	12 (44.4%)
Duration of symptoms	Months 33 (IQR 14–53) Years 2 (IQR 1–4)	Months 20 (IQR12–29.25) Years 1(IQR 1–2.25)
Time to follow-up	Months 101(IQR 88.5–131.8) Years 8 (IQR 7–10.75)	Months 122 (IQR 98–163) Years 10 (IQR 8–13)

**Table 2:** Outcome of the Ogilvie–Harris score

	Pain	Swelling	Stiffness	Limping	Activity
	*N* = 28 (100%)	*N* = 28 (100%)	*N* = 27 (100%)	*N* = 28 (100%)	*N* = 28 (100%)
Excellent	15 (54%)	17 (61%)	15 (56%)	20 (71%)	14 (50%)
Good	3 (11%)	6 (21%)	3 (11%)	1 (4%)	6 (21%)
Fair	6 (21%)	2 (7%)	9 (33%)	4 (14%)	5 (18%)
Poor	4 (14%)	3 (11%)	–	3 (11%)	3 (11%)

In 11 (39%) of the patients, complaints never reoccurred and in 6 (21%) patients, there were minor residual complaints. Eight (29%) patients experienced a temporary effect and three (11%) stated that the operation was ineffective. In 23 (82.1%) of the patients, their current function is (almost) normal.

In retrospect, 17 (61%) of the patients would undergo endoscopic treatment again under the exact same circumstances, 8 (29%) of the patients doubted if they would undergo surgical treatment again and 3 (11%) of the patients would prefer not to undergo this procedure again. Of the three patients that would not undergo the procedure again, one patient was, however, satisfied with the procedure.

### Physical examination results

Seventeen patients visited the outpatient clinic for a physical examination. During physical examination, dorsiflexion and plantarflexion of the affected side were similar to the unaffected side. The median AOFAS score was 100 (IQR 89–100). Two patients (11.8%) had some swelling, six patients (11.8%) had tenderness on palpation of the insertion of the Achilles tendon onto the calcaneus, and one patient (5.9%) could not walk on heels, because this was too painful.

### Radiologic measurements

Table 3 presents the median PLL and PFA preoperative, 2 weeks postoperative and at follow-up. The median PPL difference between before the operation and 2 weeks after the operation was − 4 mm (IQR-6 and − 1) and the median PPL difference between 2 weeks after the operation and at follow-up was + 1 mm (0–2). None of the radiological measurements was found to be significantly associated with clinical outcome or satisfaction.

**Table 3:** The median PLL and PFA preoperative, 2 weeks postoperative and at follow-up

	Preoperative	Two weeks postoperative	At follow-up
PPL (mm)*	0 (0–3)	− 2 (− 3.5–0.75)	− 1 (− 2.5–0.5)
PFA (degrees)*	65 (63–69)	66.5 (60.75–70.25)	64 (60.75–65.25)

*Median (IQR)

### Re-operation

In two (7.1%) patients, re-operation for recurrence of symptoms was performed. The time between the first operation and re-operation was 15 months and 81 months. The first case showed a hypertrophic scar that impinged on the posterolateral edge of the calcaneus and the second case had medial and lateral complaints and an increase of the PPL with 1 mm. After re-operation, both patients had an uneventful recovery and no persisting complaints at late follow up.

### Complications

There was temporary hypoesthesia of the skin in three (10.7%) patients. No other complications occurred.

## Discussion

The most important finding of this study is that there is a high patient satisfaction and good functional outcome after endoscopic calcaneoplasty for the diagnosis of retrocalcaneal bursitis on the long term.

The overall median satisfaction score of 8.5 out of 10 is an excellent score, even as the median AOFAS score of 100. This is supported by the fact that in 82% of the patients their current function is (almost) normal and that the NRS pain scores are very low. However, the FAOS subscales are scored less than excellent which can be due to the fact that 39% of the patients are completely free of symptoms and that 21% was largely free of symptoms. Unfortunately, the response rate of 51% is low, however, the obtained results are of the first patients of this new surgical technique at the time. Therefore, this case series contributes to knowledge, since there is a paucity on the literature about the long-term outcome after endoscopic calcaneoplasty.

Nowadays, endoscopic treatment is considered to be superior to open intervention for retrocalcaneal bursitis [28]. A review by Wiegerinck et al. evaluated 397 open surgical procedures and reported a good to excellent patient satisfaction of 77% [2, 17, 19, 28]. In total, 150 endoscopic procedures were evaluated and reported a good-to-excellent patient satisfaction of 90%. Possible reasons are quick resumption of activities, better cosmetic outcome and less complications [17, 28].

To our knowledge, the longest follow-up study for endoscopic calcaneoplasty results is published by Kaynak et al. after an average time of 58 months [13]. Their study included 30 feet of 28 patients and state that all patients were satisfied with the results; however, this was based on the question if patients were satisfied at the final follow-up visit. The degree of satisfaction was not measured and a face-to-face interview and can result in bias. The average AOFAS score improved from 52.6 (range 24–75) preoperative to 98.6 (range 90–100) postoperative. Our study shows similar results with a median AOFAS score of 100 (IQR 89–100). Jerosch et al. published three studies on their cohort in 2003, 2007 and 2012 [9–11]. In 2012, the average follow-up is 46.3 (range 8–120) months in 164 patients. The clinical examination was performed using the Ogilvie–Harris score of which several parameters were documented: 71 patients presented good and 84 patients excellent results, while five patients showed fair results and four patients only poor results. However, we cannot compare our Ogilvie–Harris score, since there are five subscores in the Ogilvie–Harris score, thereby it is a self-reported score by the patient and not a clinical examination score by the doctor. Ortmann reported on 30 heels in 28 patients with a mean follow-up of 35 months (range 3–62). The preoperative AOFAS was filled out retrospective with a mean 62 points (36–77) and postoperative score was 97 (78–100), similar to previous results [17].

In 17 (60%) of the patients, complaints never reoccurred or had minor residual complaints and 23 (82%) had a current function that was (almost) normal, this corresponds to the fact that 17 (61%) would undergo undergo endoscopic treatment again under the exact same circumstances. However, it cannot be explained that eight (29%) of the patients doubted if they would undergo surgical treatment again and that of these patients, five had no reoccurring complaints or had minor residual complaints.

In the previous endoscopic studies, Kaynak et al. reports no complications, Jerosch et al. had 1 superficial inflammation of the skin (0.6%) in the cohort of 164 patients and Ortmann et al. reported an Achilles tendon rupture 3 weeks after operation (3.3%) and 1 reoperation (3.3%) because of residual pain and swelling directly postoperative [11, 13, 17]. In line with literature, only minor complications occurred in our case series namely temporary hypoesthesia of the skin in three patients (10.7%). There were two re-operations (7.1%), one was due to hypertrophic scarring and the other patient was without symptoms for 7 years after the first operation. After recurrence of the complaints, a subsequent endoscopic calcaneoplasty was performed with an uneventful recovery.

Concerning radiography, it was stated that the Fowler and Philip angle can be measured, and that an angle of 75° or more would cause symptoms [5]. However, other authors found a poor relationship between the FPA and symptoms [4, 14]. Our study shows a PFA of 65° at baseline, which also disagrees with that statement. The exostosis is located on top of the calcaneus and when operated on, this will not influence the FPA. Thus, angle measurements on a lateral radiograph are considered to be obsolete. The height of the calcaneal prominence is more significant than angle measurements. Pavlov realised this and developed the parallel-pitch lines measurement [20]. During operation, we resected 4 mm (IQR 6–1) and at the median follow-up time, there was growth of 1 mm, however, this is within the measurement error and is, therefore, negligible.

Due to the design of this study, it was not possible to compare the results at follow-up with pre-operative data, which can cause inherent bias. To that end, it is uncertain to what extend the functional outcome scores improved due to surgical treatment. Also, there is a low response rate of 51% and this small number of patients is a limitation. Every effort is made to increase our response rate, however, this study regards patients that were treated 9–19 years ago. In the meantime, patients had moved and the addresses were unknown. Also, our department is specialized in foot and ankle pathology, hereby six of the patients visited from abroad for treatment and could not be contacted which resulted in a lower response rate.

Outcome of short-term endoscopic calcaneoplasty shows promising results on patients’ satisfactory, pain scale and the different scales used to assess patient outcome. To our knowledge, this is the first study to report on long-term outcome of patients treated by endoscopic calcaneoplasty for retrocalcaneal bursitis. This study contributes to a better knowledge on long-term outcome of endoscopic calcaneoplasty and will aid orthopedic surgeons in counselling future patients affected by retrocalcaneal bursitis.

## Conclusion

Despite the limited response rate, this study shows high patient satisfaction and good long-term functional outcome in patients affected by retrocalcaneal bursitis who underwent endoscopic calcaneoplasty.

## References

[1] Aaron DL, Patel A, Kayiaros S, Calfee R (2011). Four common types of bursitis: diagnosis and management. J Am Acad Orthop Surg.

[2] Angermann P (1990). Chronic retrocalcaneal bursitis treated by resection of the calcaneus. Foot Ankle.

[3] Boffeli TJ, Peterson MC (2012). The Keck and Kelly wedge calcaneal osteotomy for Haglund’s deformity: a technique for reproducible results. J Foot Ankle Surg.

[4] Bulstra GH, van Rheenen TA, Scholtes VA (2015). Can we measure the heel bump? Radiographic evaluation of Haglund’s deformity. J Foot Ankle Surg.

[5] Fowler A (1945). Abnormality of the calcaneus as a cause of painful heel. Its diagnosis and operative treatment. Br J Surg.

[6] Haglund P (1928). Beitrag zur Klinik der Achillessshne. Arch Orthop Chir.

[7] Holdgate A, Asha S, Craig J, Thompson J (2003). Comparison of a verbal numeric rating scale with the visual analogue scale for the measurement of acute pain. Emerg Med (Fremantle).

[8] Jerosch J (2015). Endoscopic calcaneoplasty. Foot Ankle Clin.

[9] Jerosch J, Nasef NM (2003). Endoscopic calcaneoplasty–rationale, surgical technique, and early results: a preliminary report. Knee Surg Sports Traumatol Arthrosc.

[10] Jerosch J, Schunck J, Sokkar SH (2007). Endoscopic calcaneoplasty (ECP) as a surgical treatment of Haglund’s syndrome. Knee Surg Sports Traumatol Arthrosc.

[11] Jerosch J, Sokkar S, Ducker M, Donner A (2012). Endoscopic calcaneoplasty (ECP) in Haglund’s syndrome. Indication, surgical technique, surgical findings and results. Z Orthop Unfall.

[12] Kachlik D, Baca V, Cepelik M, Hajek P, Mandys V, Musil V (2008). Clinical anatomy of the retrocalcaneal bursa. Surg Radiol Anat.

[13] Kaynak G, Ogut T, Yontar NS, Botanlioglu H, Can A, Unlu MC (2013). Endoscopic calcaneoplasty: 5-year results. Acta Orthop Traumatol Turc.

[14] Keck SW, Kelly PJ (1965). Bursitis of the posterior part of the heel; evaluation of surgical treatment of eighteen patients. J Bone Joint Surg Am.

[15] Kitaoka HB, Alexander IJ, Adelaar RS, Nunley JA, Myerson MS, Sanders M (1994). Clinical rating systems for the ankle-hindfoot, midfoot, hallux, and lesser toes. Foot Ankle Int.

[16] Lamers LM, Stalmeier PF, McDonnell J, Krabbe PF, van Busschbach JJ (2005). Measuring the quality of life in economic evaluations: the Dutch EQ-5D tariff. Ned Tijdschr Geneeskd.

[17] Ortmann FW, McBryde AM (2007). Endoscopic bony and soft-tissue decompression of the retrocalcaneal space for the treatment of Haglund deformity and retrocalcaneal bursitis. Foot Ankle Int.

[18] Pasa L, Kuzma J, Herufek R, Prokes J, Sprlakova-Pukova A (2018). Arthroscopic treatment of chronic retrocalcaneal bursitis—endoscopic calcaneoplasty. Acta Chir Orthop Traumatol Cech.

[19] Pauker M, Katz K, Yosipovitch Z (1992). Calcaneal ostectomy for Haglund disease. J Foot Surg.

[20] Pavlov H, Heneghan MA, Hersh A, Goldman AB, Vigorita V (1982). The Haglund syndrome: initial and differential diagnosis. Radiology.

[21] Sella EJ, Caminear DS, McLarney EA (1998). Haglund’s syndrome. J Foot Ankle Surg.

[22] Sierevelt IN, Beimers L, van Bergen CJ, Haverkamp D, Terwee CB, Kerkhoffs GM (2015). Validation of the Dutch language version of the foot and ankle outcome score. Knee Surg Sports Traumatol Arthrosc.

[23] Sierevelt IN, van Eekeren IC, Haverkamp D, Reilingh ML, Terwee CB, Kerkhoffs GM (2016). Evaluation of the Dutch version of the foot and ankle outcome score (FAOS): responsiveness and minimally important change. Knee Surg Sports Traumatol Arthrosc.

[24] Sofka CM, Adler RS, Positano R, Pavlov H, Luchs JS (2006). Haglund’s syndrome: diagnosis and treatment using sonography. HSS J.

[25] van Dijk CN, Scholten PE, Krips R (2000). A 2-portal endoscopic approach for diagnosis and treatment of posterior ankle pathology. Arthroscopy.

[26] van Dijk CN, van Dyk GE, Scholten PE, Kort NP (2001). Endoscopic calcaneoplasty. Am J Sports Med.

[27] Weel H, Zwiers R, Sierevelt IN, Haverkamp D, van Dijk CN, Kerkhoffs GM (2015). Dutch-language patient-reported outcome measures for foot and ankle injuries; a systematic review. Ned Tijdschr Geneeskd.

[28] Wiegerinck JI, Kok AC, van Dijk CN (2012). Surgical treatment of chronic retrocalcaneal bursitis. Arthroscopy.

